# Predictive modeling for peri-implantitis by using machine learning techniques

**DOI:** 10.1038/s41598-021-90642-4

**Published:** 2021-05-27

**Authors:** Tomoaki Mameno, Masahiro Wada, Kazunori Nozaki, Toshihito Takahashi, Yoshitaka Tsujioka, Suzuna Akema, Daisuke Hasegawa, Kazunori Ikebe

**Affiliations:** 1 Department of Prosthodontics, Gerodontology and Oral Rehabilitation, Osaka University Graduate School of Dentistry, 1-8 Yamadaoka, Suita, Osaka 565-0871 Japan; 2 Division for Medical Information, Osaka University Dental Hospital, Suita, Japan

**Keywords:** Dental diseases, Outcomes research

## Abstract

The purpose of this retrospective cohort study was to create a model for predicting the onset of peri-implantitis by using machine learning methods and to clarify interactions between risk indicators. This study evaluated 254 implants, 127 with and 127 without peri-implantitis, from among 1408 implants with at least 4 years in function. Demographic data and parameters known to be risk factors for the development of peri-implantitis were analyzed with three models: logistic regression, support vector machines, and random forests (RF). As the results, RF had the highest performance in predicting the onset of peri-implantitis (AUC: 0.71, accuracy: 0.70, precision: 0.72, recall: 0.66, and f1-score: 0.69). The factor that had the most influence on prediction was implant functional time, followed by oral hygiene. In addition, PCR of more than 50% to 60%, smoking more than 3 cigarettes/day, KMW less than 2 mm, and the presence of less than two occlusal supports tended to be associated with an increased risk of peri-implantitis. Moreover, these risk indicators were not independent and had complex effects on each other. The results of this study suggest that peri-implantitis onset was predicted in 70% of cases, by RF which allows consideration of nonlinear relational data with complex interactions.

## Introduction

Peri-implantitis is an inflammatory disease in peri-implant tissues mainly caused by plaque accumulation, which results in bone loss around the implant^1,2^. Although the reported prevalence varies, approximately 10% to 20% of implants develop peri-implantitis^3,4^. Many studies have used statistical analyses to examine the risk indicators for peri-implantitis. Heitz-Mayfield^5^ reported that a history of periodontitis, oral hygiene status, and smoking were risk indicators for peri-implantitis onset. Several studies have reported that lack of regular maintenance visits, diabetes, implant surface characteristics, and excess cement are related to peri-implantitis^6–8^. Other studies have reported that the presence of keratinized tissue and the number of occlusal supports correlate with peri-implantitis development^9,10^. Although this evidence is helpful in clinical practice, many aspects regarding the degree of influence of each factor, the detailed mechanisms, and the causal relationships have not been clarified. Moreover, preoperative evaluation based on this evidence is expected to predict and prevent the onset of peri-implantitis.

Statistical interaction may arise in medical statistics when there are two or more independent variables and the effect of one variable on the outcome depends on the value of another variable. If a simple interaction between binary variables or a linear interaction is present, the interaction can be examined by conducting a statistical analysis that includes interaction terms. However, such a simple relationship is rarely seen in clinical medicine. Support vector machines (SVM) and random forests (RF) are effective methods for data analysis with complex interactions^11^. These binary classification models use the machine learning (ML) method, which belongs to the domain of artificial intelligence. In general, logistic regression analysis (LR), which is widely used as a binary classification model, analyzes data depending on the Bernoulli distribution. Because the distribution is specified, relatively high prediction performance can be achieved even with a small number of samples. However, there are limits to the analysis of nonlinear relational data with complex interactions. In contrast, ML techniques, such as SVM and RF, make it possible to discriminate complicated nonlinear patterns without prior assumptions about the probability distribution of the data^12^. These methods can be an important component of approaches to estimate causal effects in observational studies, with good performance in reducing bias and controlling for confounding^13^. Although these ML models have been widely used in medicine for image detection, diagnosis, and outcome prediction^14^, there have been no reports on the construction of a model for predicting the onset of peri-implantitis and for interpreting its nonlinear relationships.

The purpose of this study was to construct a model for predicting the onset of peri-implantitis by using the ML classification method, which is robust to the bias and collinearity of variables, and to clarify the relationships between risk indicators.

## Results

**Figure 1:**
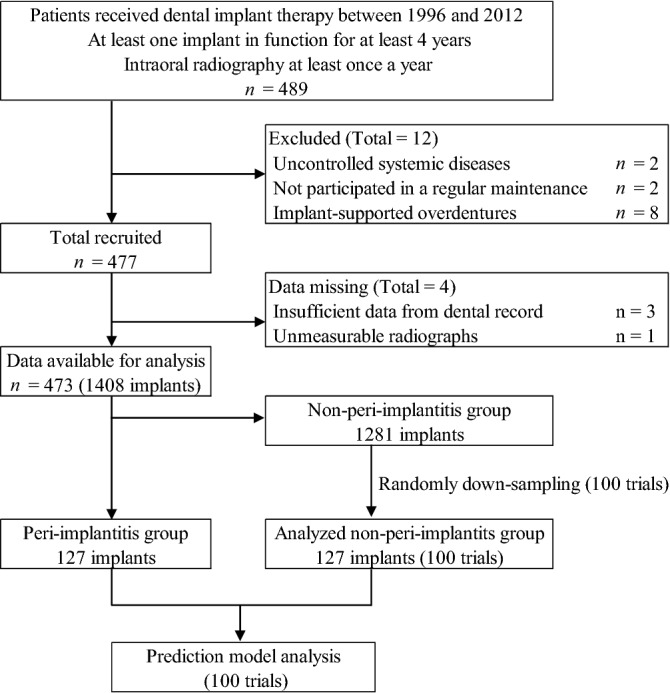
Flow diagram outlining the inclusion and exclusion criteria and study design. This figure was created with Excel 2019 version 1808 (Microsoft, Inc, Redmond, WA).

Of the 489 patients recruited for this study, after application of the exclusion criteria, 16 patients were excluded. Finally, this study evaluated 473 patients (314 women, 159 men) who were treated with 1408 implants manufactured by 6 brands (608 Nobel Biocare, 597 Dentsply, 122 Zimmer biomet, 56 Straumann, 22 GC, 3 Camlog). 127 implants (9.0%) were classed into peri-implantitis group (PI-group). 1281 implants had less than 1 mm of bone resorption, were classified into the non-peri-implantitis group (Non-PI-group). After down-sample adjustment, 254 implants were analyzed by ML models (Fig. 1).

All original data variables for the implants and analyzed data are presented in Table 1.

**Table 1:** Characteristics of evaluated and analyzed implants. Average of non-PI-group show the mean value of non-PI in the analyzed population obtained from 100 trials. SD: standard deviation, PCR: plaque control record, OS: number of occlusal supports, KMW: keratinized mucosa width.

Variables		Original data (n = 1408)				Analyzed data	
		PI-group		Non-PI-group		Average of non-PI-group (n = 127*100 trials)	
Categorical variables		n	%	n	%	n/100	%
Total		127		1281		127	
Sex	Men	56	44.1	450	35.1	43.4	34.2
	Women	71	55.9	831	64.9	83.6	65.8
History of periodontitis	No	53	41.7	621	48.5	61.6	48.5
	Yes	74	58.3	660	51.5	65.4	51.5
Implant position	Mandible	70	55.1	564	44.0	56.3	44.3
	Maxilla	57	44.9	717	56.0	70.7	55.7
Fixation method	Screw	90	70.9	928	72.4	91.7	72.2
	Cement	37	29.1	353	27.6	35.3	27.8

Continuous variables		Mean	SD	Mean	SD	Mean	SD
Age	(years)	67.06	11.75	65.77	10.41	65.75	10.51
PCR	(%)	30.20	22.46	22.06	17.91	21.98	17.84
Smoking	(cigarettes)	3.98	8.98	1.99	6.49	1.90	6.34
OS	(unit)	5.91	4.32	7.40	3.85	7.37	3.88
Functional time	(months)	62.58	32.05	70.66	29.86	70.92	29.78
KMW	(mm)	1.52	1.50	2.39	1.74	2.37	1.73
Bone loss	(mm)	1.71	0.77	0.09	0.44	0.09	0.45

**Figure 2:**
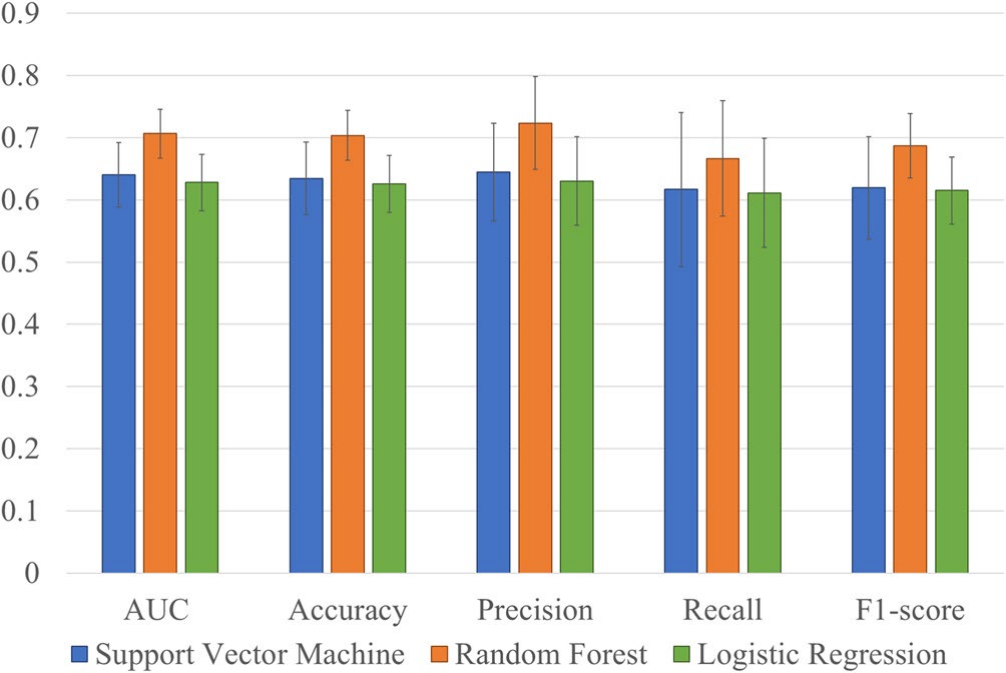
Performance metrics of each classification model. The error bars represent standard deviation. AUC: area under the receiver operating characteristic curve. This figure was created with Excel 2019 version 1808 (Microsoft, Inc, Redmond, WA).

Non-PI implants analyzed by ensemble averaging of 100 trials showed a sample distribution similar to the original non-PI-group. The mean performance metrics obtained with 100 times prediction model analyses in each classifier are shown in Fig. 2.

**Figure 3:**
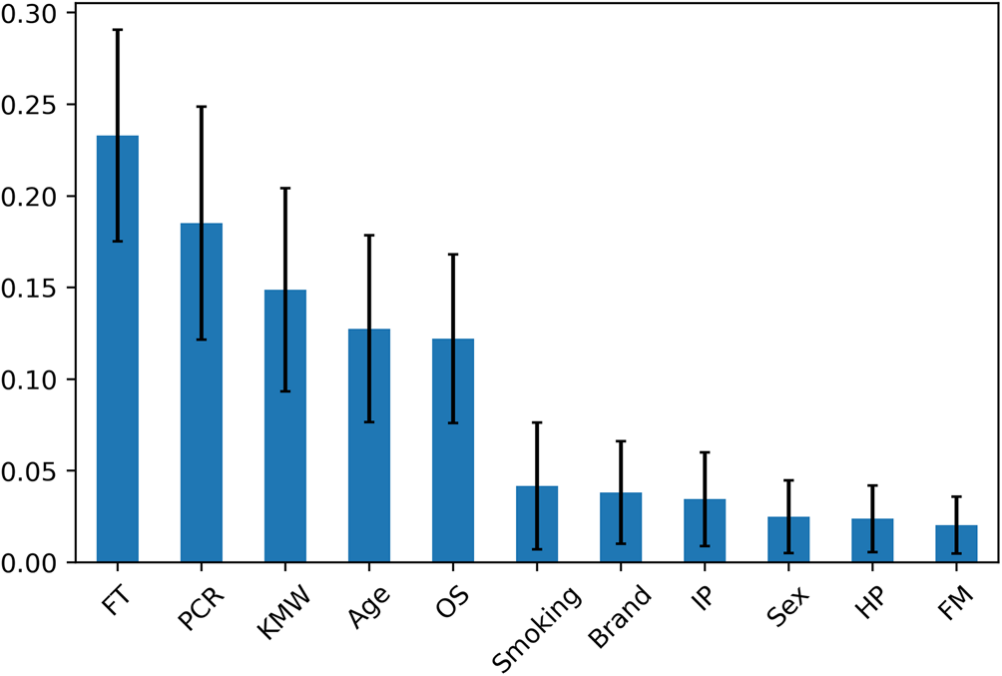
Mean values of feature importance in the RF prediction model. The error bars represent standard deviation. FT: functional time, PCR: plaque control record, KMW: keratinized mucosa width, OS: number of occlusal supports, IP: implant position, HP: history of periodontitis and FM: fixation method. This figure was created with Python version 3.7.7 (Python Software Foundation, Beaverton, OR).

The mean area under the receiver operating characteristic curve (AUC) values (± SD) for SVM, RF, and LR were 0.64 (0.05), 0.71 (0.04), and 0.63 (0.05), respectively. RF showed the highest performance for prediction of peri-implantitis (accuracy: 0.70, precision: 0.72, recall: 0.66, and f1-score: 0.69). Figure 3 shows the values of feature importance (± SD) in the RF prediction model.

The values in descending order were functional time (0.232 ± 0.058), plaque control record (PCR; O’Leary score) (0.185 ± 0.064), keratinized mucosa width (KMW) (0.149 ± 0.055), age (0.127 ± 0.051), number of occlusal supports (0.122 ± 0.046), number of cigarettes smoked (0.042 ± 0.035), brand (0.038 ± 0.028), implant position (0.034 ± 0.026), sex (0.025 ± 0.019), history of periodontitis (0.024 ± 0.018), and fixation method (0.020 ± 0.016).

**Figure 4:**
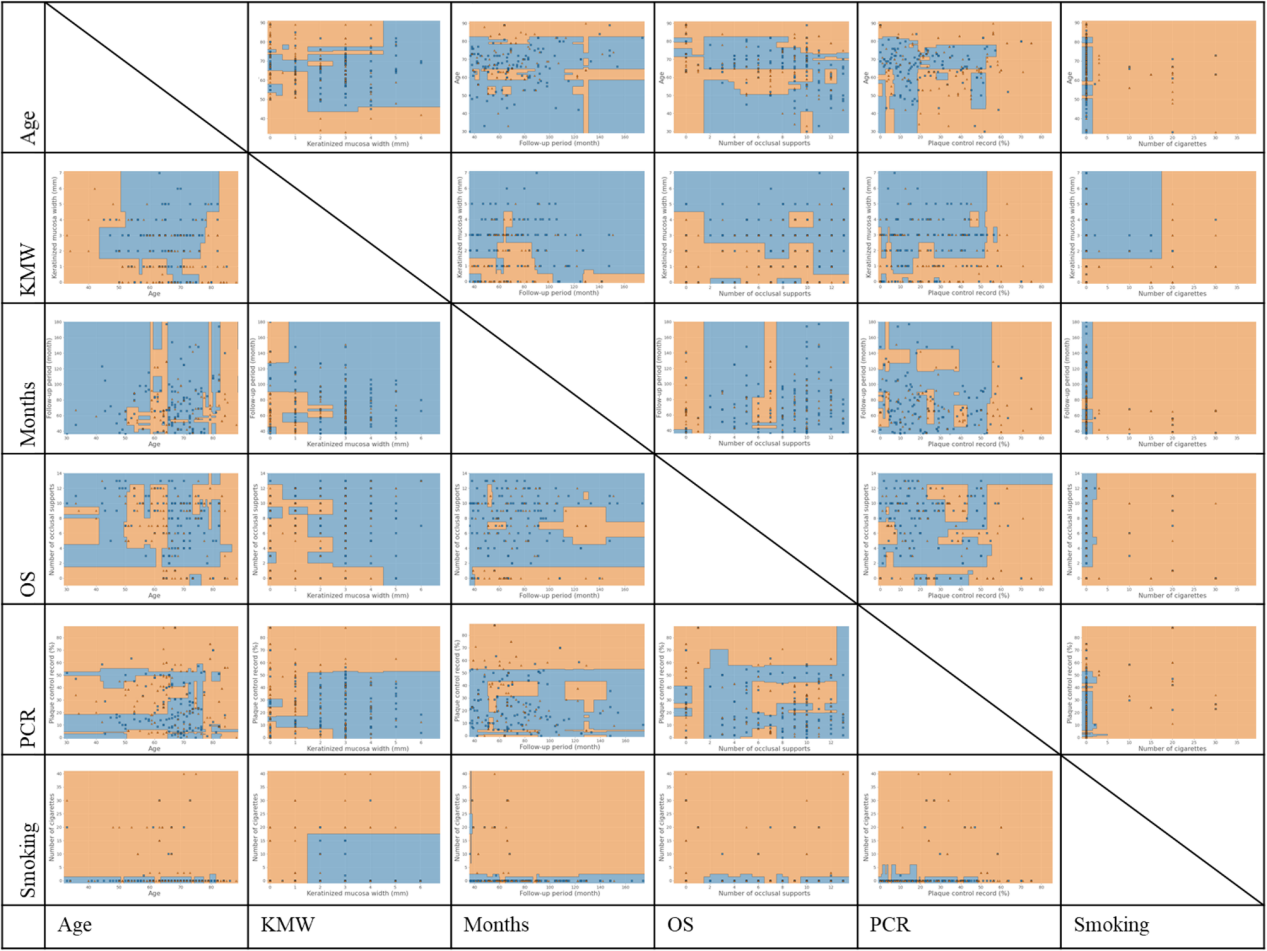
Classification diagrams for predicting onset of peri-implantitis with RF. Testing data that fall in orange areas are predicted to be peri-implantitis and those that fall in blue areas are predicted to be non-peri-implantitis. PCR: plaque control record, OS: number of occlusal supports, KMW: keratinized mucosa width. This figure was created with Python version 3.7.7 (Python Software Foundation, Beaverton, OR) and PowerPoint 2019 version 1808 (Microsoft, Inc, Redmond, WA).

Figure 4 shows the decision boundaries of the RF, which showed the highest performance, and Appendix 1 and 2 show those of the SVM and LR.

Testing data that fell in orange areas were predicted to be PI-group and those that fell in blue areas were predicted to be non-PI-group. A general feature of the classification diagram of the RF was that the form of the decision boundaries obtained by the optimization of hyperparameters was nonlinear. The mean values of each best hyperparameters of RF tuned by 100 times group fivefold cross-validations were as follows: maximum depth was 5.1, minimum sample leaf was 4.8, minimum sample split was 6.0, number of estimators was 10.0. In addition, in the classification diagrams with age on the horizontal axis (Fig. 4, first column from the left), there was inconsistent distribution bias resulting from the variable on the vertical axis. In the diagrams with KMW on the horizontal axis (Fig. 4, second column from the left), the right side was classified as non-PI-group with a boundary of 1 to 2 mm. Peri-implantitis was diagnosed in consistent distribution, regardless of the variable on the vertical axis, in diagrams of the functional time (Fig. 4, third column from the left). Regarding the number of occlusal supports (Fig. 4, fourth column from the left), those with one or fewer occlusal supports tended to be classified as having peri-implantitis. In the diagrams for the PCR (Fig. 4, fifth column from the left), many on the right side were classified as having peri-implantitis, with a boundary of approximately 50% to 60%. For smoking habits (Fig. 4, sixth column from the left), the higher the number of daily cigarettes, the greater the percentage of peri-implantitis; the appearance of classification changed at approximately 3 cigarettes with the exception of classification diagram with KMW on the vertical axis.

## Discussion

In this retrospective cohort study, various risk indicators for the onset of peri-implantitis were evaluated in implants with at least 4 years in function from final prosthesis delivery. LR, SVM, and RF were used to predict the onset of peri-implantitis using these risk indicators, and the accuracy of each analysis was investigated. The results showed that the RF produced the most accurate predictions. In addition, the AUC, which is the performance indicator for binary classification, was relatively high for the RF. Interestingly, all classification patterns of the RF and SVM showed complex nonlinear aspects.

Several studies have examined risk indicators for peri-implantitis in detail. However, there have been no reports on the use of ML methods to predict the onset of peri-implantitis. The present study is the first attempt to investigate the onset of peri-implantitis by using ML and applying the findings accumulated with statistical analysis; this method is considered a novel approach. Regarding the analysis method, it is common to perform the analysis while considering the issue of clustered samples when searching for factors from the observed results by statistical analysis^9,10^. On the other hand, this study attempts to predict peri-implantitis from given risk indicators in machine learning that does not assume prior distribution. Therefore, LR was used instead of multi-level analysis represented by mixed effect model and generalized estimation equation. In addition, SVM and RF were selected as machine learning models with easy interpretation, because of advantages in the ability to calculate the relative importance of each feature with respect to the model outcome and to draw the classification diagram. Present study collected single or multiple implant data from the same individual. Therefore, subject-level predictions were performed with the group k-fold cross-validation approach.

This study revealed that RF had the highest classification performance among LR, SVM, and RF, which are mainly used for binary classification. Among the variables in the RF model, implant functional time was the most important for prediction of peri-implantitis, followed by PCR, KMW, age, number of occlusal supports, number of cigarettes smoked, brand, implant position, sex, history of periodontitis, and fixation method. Many studies have shown that poor oral hygiene and a history of periodontitis have a negative effect on the health of peri-implant tissue^15,16^, consistent with the results of this study. However, whether the other variables are risk factors for peri-implantitis remains debatable and no clear conclusion has been reached.

In this study, we considered the interactions between continuous variables by using nonlinear pattern classification diagrams (Fig. 4) obtained with RF, which showed the highest predictive performance. First, in the diagrams with age on the horizontal axis, an unexplained unbalanced bias was observed, regardless of the variable on the vertical axis. The influence of age on the onset of peri-implantitis is considered low because it is possible to draw a clear boundary in the diagram between factors that have an important influence on prediction. In the diagrams with KMW on the horizontal axis, the aspects of classification with clear boundaries of 1 to 2 mm were shown. This finding indicates that the KMW is an important predictor of the onset of peri-implantitis. Additionally, there might be a certain amount of KMW that is sufficient (2 mm in the figure). A cut-off value of 2 mm is often used when discussing KMW as a risk factor^17^. It is very interesting that the classification used in past reports falls in line with the results of this study. Next, regarding the functional time, small mass of PI-group in the right side of diagrams shows that those with longer functional time tended to be classified as having peri-implantitis. However, it was impossible to draw a clear boundary in the diagram between months and the other factors. This indicates that it is difficult to predict the onset of peri-implantitis based on the functional time alone. As other studies have reported^15,18^, a non-linear and accelerating pattern of peri-implantitis onset may influence this result. Regarding the number of occlusal supports, the greater the number of occlusal supports, the lower the risk of peri-implantitis in this study. This finding is consistent with that reported by Mameno, et al.^9^. In addition, implants with fewer than two occlusal supports tended to have a higher risk of peri-implantitis in this study. In the PCR diagrams, the risk of developing peri-implantitis was high on the right side of the 50% to 60% line. Finally, regarding smoking, the higher the number of cigarettes, the higher the risk of peri-implantitis, with the risk changing at about 3 cigarettes per day. Additionally, focusing on the figure with KMW on the vertical axis, a clear boundary region surrounded by the lines of 15 to 20 cigarettes smoked and 1 to 2 mm of KMW is drawn. Although the number of smokers included in this study is small to draw clear conclusions, there may be a strong association between keratinized mucosa and smoking.

Because these classification diagrams extract only two variables to describe an original multidimensional classifying space in two dimensions, detailed values should not be discussed. However, all classification diagrams showed that each factor would be not independent. The ML models have advantages in the analysis of data with interactions^13^. For example, in the classification diagram with KMW on the horizontal axis and PCR on the vertical axis (Fig. 4, second column from left, second row from bottom), the RF has a steep boundary line slope in the area of 1 to 2 mm, and a very gentle slope to the right of that. In contrast, the classification boundary line in the LR diagram was a straight line with a constant slope with the origin near 0 (Appendix 1, second column from left, second row from bottom). This discrepancy indicates the difference in the classification pattern between the lower left area (implants with less KMW and lower PCR) and the upper right area (implants with more KMW and higher PCR) in the diagram. In other words, LR tended to misclassify peri-implantitis as non-PI-group in the lower left and upper right areas, and similarly misclassified non-PI-group as having peri-implantitis in the central area. If there were no interaction between PCR and KMW, the boundary line drawn by the RF would be linear and consistent with that of the LR analysis. This explains why the AUC of LR was lowest among the models.

There are several limitations in this study. First, only participants who satisfied the inclusion criteria were targeted in this study, which may have resulted in selection bias. Good patient compliance for treatment might result that heavy smokers or those with extremely poor oral hygiene were not included in this study. Next, the progression of peri-implantitis did not evaluate and remained unclear, because this study focused on the risk indicators for peri-implantitis onset. Additionally, the variables shown as risk indicators were used for prediction in this study. In order to make predictions with higher accuracy, it is necessary to include factors considered to be clinically important, such as patient cleaning habits, superstructure morphology, and implantation depth and so on. Despite the limitations outlined above, the first attempt to predict the onset of peri-implantitis by using ML are of clinical importance. The application of the ML method could enable prediction of the onset of peri-implantitis with greater accuracy than other methods and could lead to new discoveries.

In conclusion, peri-implantitis onset was predicted by RF in 70% of cases. The factor that had the most influence on prediction was implant functional time, followed by oral hygiene. In addition, PCR of more than 50% to 60%, smoking more than 3 cigarettes/day, KMW less than 2 mm, and the presence of less than two occlusal supports tended to be associated with an increased risk of peri-implantitis. Moreover, these risk indicators were not independent and had complex effects on each other.

## Materials and methods

### Patients and collected data

Participants in this study received dental implant therapy between November 1996 and December 2012 at a dental university hospital or at one of seven general dental offices. After the purpose of this study was explained, all patients who were willing to take part in the study provided informed consent. Inclusion criteria were as follows: presence of at least one titanium implant in function for at least 4 years and follow-up with intraoral radiography at least once a year. Patients with uncontrolled systemic disease, those who did not participate in a regular maintenance program more than once per year, and those who received removable dental implant prostheses were excluded. In this retrospective cohort study, each implant was evaluated at baseline and during the follow-up period. Baseline was defined as a point in time 1 year after delivery of the final prosthesis in consideration of bone remodeling following prosthesis insertion; follow-up was defined as more than 3 years after baseline. Evaluation items were collected from treatment records, direct interviews, and oral examination at follow-up. Each implant was evaluated for the following items, which are currently considered potential risk indicators of peri-implantitis^9,10,15^: history of periodontitis from the treatment records, defined as the presence of periodontal pockets more than 6 mm deep and attachment loss of 2 mm^20^; PCR; smoking habit; number of occlusal supports by natural teeth; jaw position (maxilla or mandible); fixation method (cement or screw); and KMW around each implant. In carrying out this study, all the doctors had several meetings to calibrate the probing measurement (unifying the type of probe and probing pressure to 15 g using electronic scale), keratinized mucosa measurement method and PCR measurement method.

### Radiographic evaluation and definition of peri-implantitis

The bone level around each implant was measured on an intraoral radiograph taken with a cone indicator (CID III, Hanshin Technical Laboratory Corp., Japan). Before measuring the bone loss on the intraoral radiographs, intra- and inter‐observer error were confirmed by using intra‐class correlation case 1 and 2 analyses. There was no significant difference in intra‐observer error (correlation coefficient = 0.996; 95% confidence interval [CI]: 0.982–1.000) or inter‐observer error (correlation coefficient = 0.994; 95% CI: 0.985–0.998). Therefore, one examiner (MW) measured the radiographs in this study. The bone level was defined as the distance between the platform of the implant and the bone crest. The implant length and the bone crest level from the apex of the implant on the intraoral radiograph (point closest to the implant apex at mesial or distal aspect) were measured with image analysis software (ImageJ 1.49v; Wayne Rasband, National Institutes of Health, Bethesda, MD, URL: https://imagej.nih.gov/ij/download.html) at baseline and follow-up. The actual implant length was used for calibration of each measurement and bone loss was measured at baseline and in the follow-up period.

Peri-implantitis was defined as the presence of bleeding on probing and/or suppuration in the follow-up period and the presence of more than 1 mm of bone resorption from the baseline measurement. The onset time of peri-implantitis, defined as the occurrence of over 1 mm of bone resorption, was also recorded between baseline and the follow-up period. This study was approved by the Osaka University Graduate School of Dentistry Ethics Committee (H28-E24). Every clinical investigation was conducted according to the principles expressed in the Helsinki Declaration. This study also followed the STROBE (Strengthening the Reporting of Observational Studies in Epidemiology) guidelines.

### Analysis

Three binary classification ML models (LR, SVM, and RF) were used for the analysis. LR is a classical statistical analysis regression model. This is a generalized linear model that estimates parameters by using the maximum likelihood method, assuming a binomial distribution. The objective of the SVM algorithm is to find a hyperplane that separates the two classes of data points in a high-dimensional space. This method can form nonlinear decision surfaces and can be coupled with the kernel function^21^. RF is a technique for classifying data by using multiple tree-based classification models that are obtained by setting a boundary line that minimizes the Gini impurity^13^. Although these models have different algorithms, they are known to perform well as binary classifiers and have been widely used to predict outcomes in clinical studies^14^.

**Figure 5:**
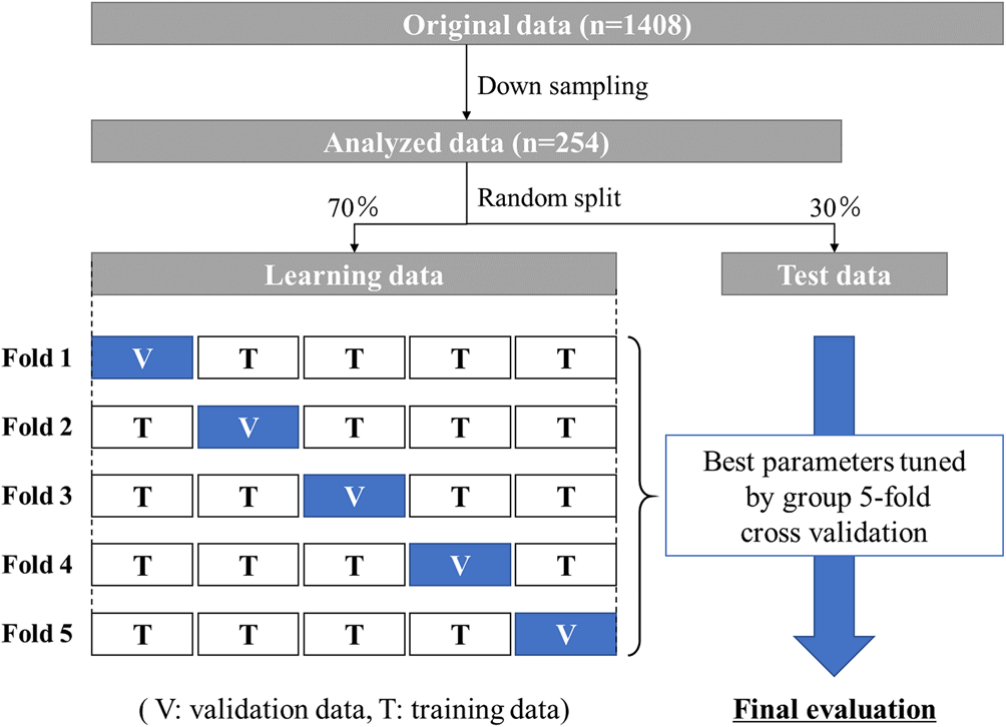
Experimental workflow of predictive machine learning approach. This figure was created with PowerPoint 2019 version 1808 (Microsoft, Inc, Redmond, WA).

The onset of peri-implantitis was selected as a target variable in these classification models, which used the basic data (age, sex, implant brand and functional time of the implant) and potential risk indicators of peri-implantitis (history of periodontitis, PCR, number of cigarettes smoked, number of occlusal supports, cement fixation, position, and KMW) as input features for prediction. After standardization of each feature, the participants were divided into two classes, the non-PI and PI groups. Because a disparity in the frequencies of the classes can have a significant negative impact on model fitting^22^, we randomly selected a subset of the non-PI group so that the class frequencies matched those of the PI group. Next, the obtained evaluation data were randomly divided into 70% learning data and 30% test data, and predictive model analysis was performed for each classification model (Fig. 5).

The learning dataset was used to provide an evaluation of a model fit while tuning hyperparameters with a grid search and group fivefold cross-validation techniques which make it possible to consider multiple samples from the same individual. A grid search technique was used to get good optimal hyperparameter values for the regularization constant and gamma in SVM, and for the maximum depth, minimum sample leaf, minimum sample split, number of estimators in RF. A grid search was performed using gridsearchCV, which is a function included in Scikit-learn. The final evaluation of the onset of peri-implantitis was performed on the remaining 30% of the test data, and the obtained predicted value and actual value were compared. The predictive performance of each model was evaluated with performance metrics (AUC, accuracy, precision, recall, and f1-score)^19^. In addition, the weight of each parameter in prediction was obtained by calculating the feature importance. To avoid sampling bias that can result from down sampling, a series of classification tasks with each ML models were performed 100 times. Finally, to understand the aspect of binary classification in each classifier, two arbitrary continuous variables were extracted and a classification diagram was drawn with the mean values of best tuned hyperparameter in 100 trials. All analyses were performed with the scikit-learn library in Python version 3.7.7 (Python Software Foundation, Beaverton, OR, URL: https://www.python.org/downloads/release/python-377/).

## Supplementary Information

Supplementary files.
