# Supplementary material for: Predictive modeling for peri-implantitis by using machine learning techniques
Source: Sci Rep. 2021 May 27;11:11090. doi: 10.1038/s41598-021-90642-4 (PMC8160334; doi:10.1038/s41598-021-90642-4)
Supplement: Supplementary file 1 — Supplementary files. [file 41598_2021_90642_MOESM1_ESM.docx]

**Title**

Predictive modeling for peri-implantitis by using machine learning techniques

Tomoaki Mameno^1^

Masahiro Wada^1^

Kazunori Nozaki^2^

Toshihito Takahashi^1^

Yoshitaka Tsujioka^1^

Suzuna Akema^1^

Daisuke Hasegawa^1^

Kazunori Ikebe^1^

1. Department of Prosthodontics, Gerodontology and Oral Rehabilitation, Osaka University Graduate School of Dentistry
2. Division for Medical Information, Osaka University Dental Hospital

**Corresponding Author**

Tomoaki Mameno, DDS, PhD, Assistant professor

Department of Prosthodontics, Gerodontology and Oral Rehabilitation, Osaka University Graduate School of Dentistry,

1-8 Yamadaoka, Suita, Osaka 565-0871, Japan

Tel: +81-6-6879-2954

Fax: +81-6-6879-2957

E-mail: [mameno@dent.osaka-u.ac.jp](mailto:mameno@dent.osaka-u.ac.jp)


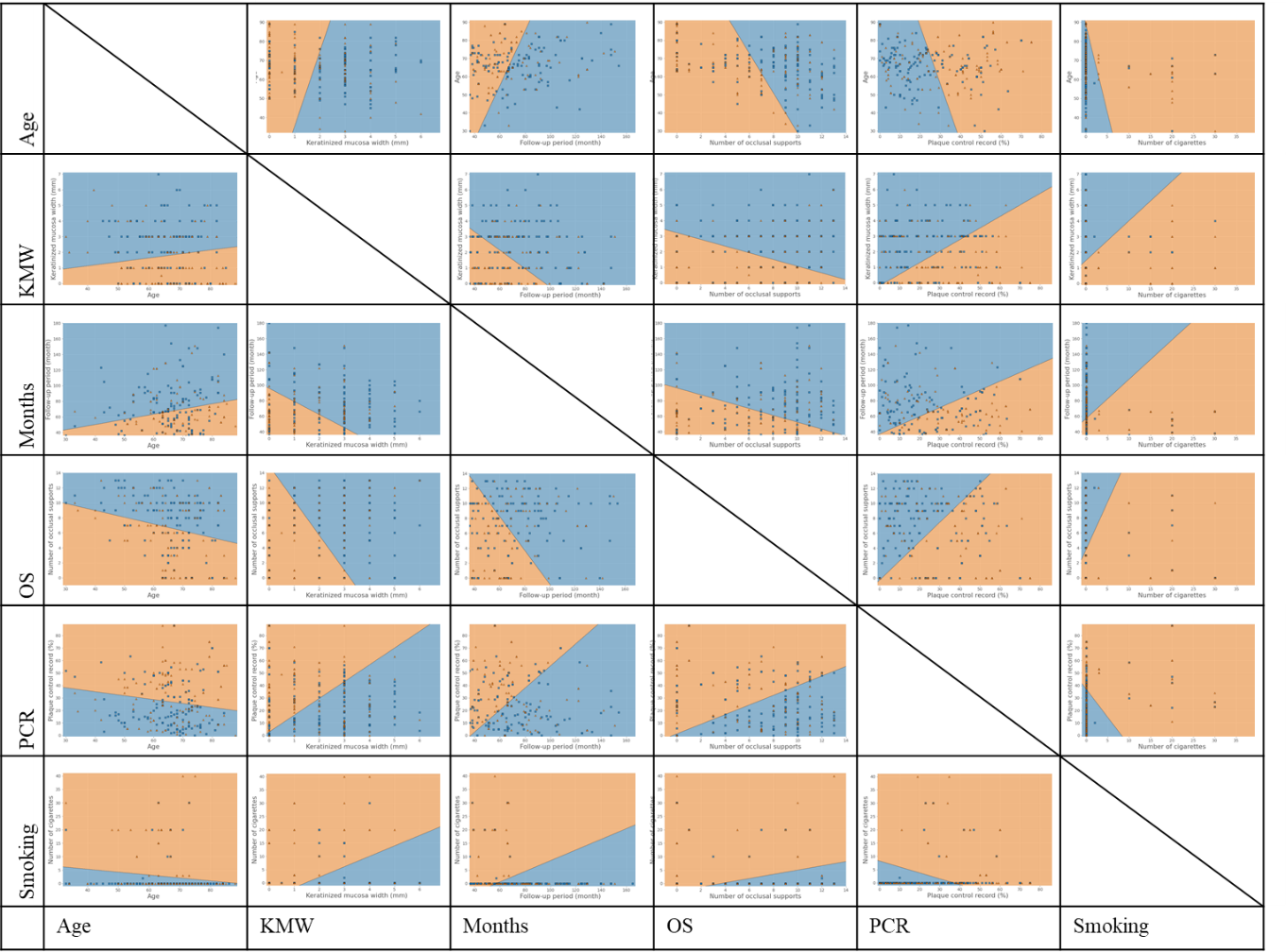


**Appendix 1.** Classification diagrams for predicting onset of peri-implantitis with LR.

Testing data that fall in orange areas are predicted to be peri-implantitis and those that fall in blue areas are predicted to be non-peri-implantitis. PCR: plaque control record, OS: number of occlusal supports, KMW: keratinized mucosa width. This figure was created with Python version 3.7.7 (Python Software Foundation, Beaverton, OR) and PowerPoint 2019 version 1808 (Microsoft, Inc, Redmond, WA).


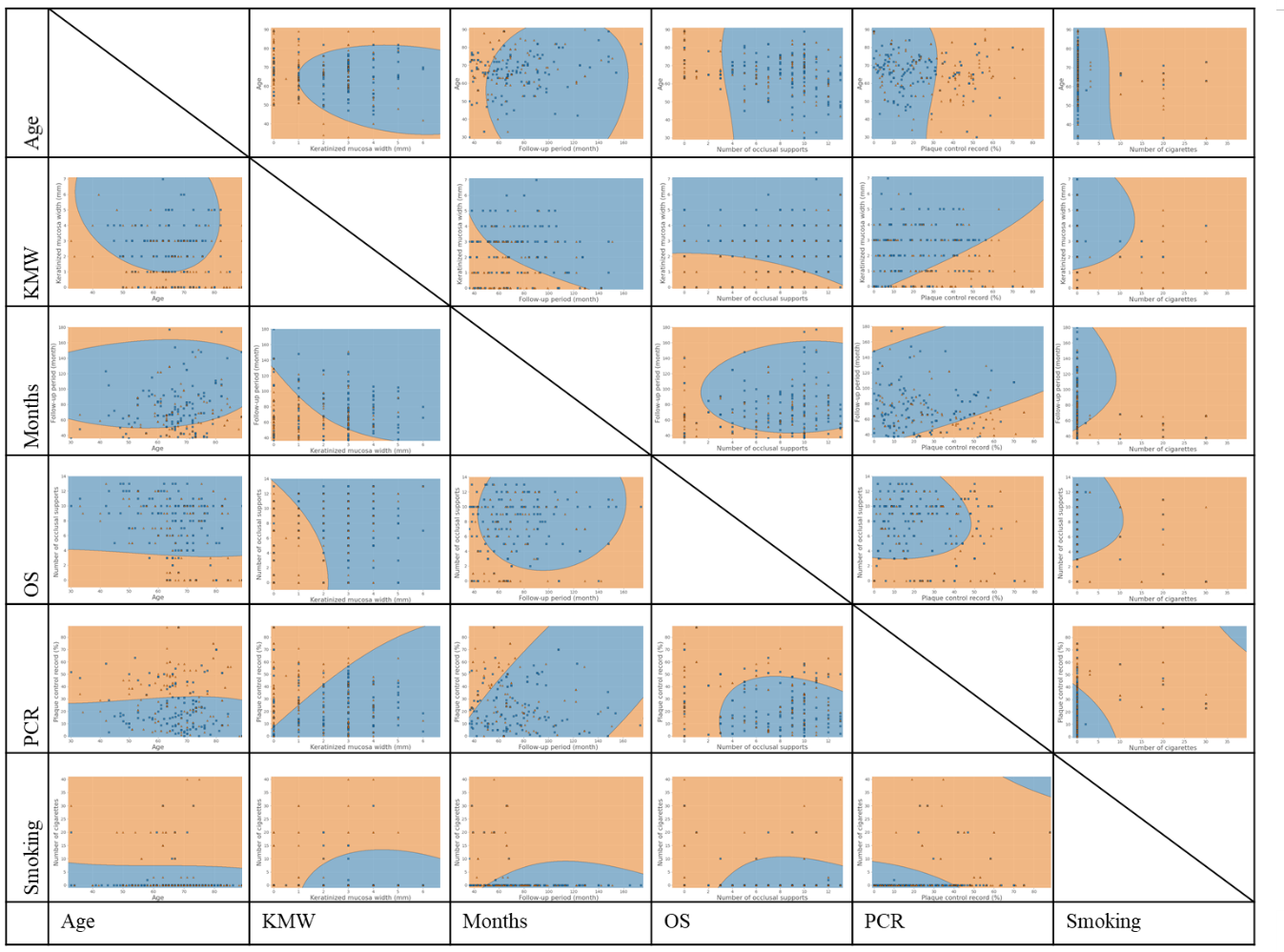


**Appendix 2.** Classification diagrams for predicting onset of peri-implantitis with SVM.

Testing data that fall in orange areas are predicted to be peri-implantitis and those that fall in blue areas are predicted to be non-peri-implantitis. PCR: plaque control record, OS: number of occlusal supports, KMW: keratinized mucosa width. This figure was created with Python version 3.7.7 (Python Software Foundation, Beaverton, OR) and PowerPoint 2019 version 1808 (Microsoft, Inc, Redmond, WA).
